# Linggui Zhugan Decoction for peripheral vertigo: A protocol for systematic review and meta-analysis

**DOI:** 10.1097/MD.0000000000025563

**Published:** 2021-04-23

**Authors:** Hongmei Ma, Liang Guo, Yong Chen, Wanning Lan, Jiyuan Zheng, Danyun Li, Ziyin Chen, Xinju Hou

**Affiliations:** a Nanchang Hongdu Hospital of Traditional Chinese Medicine; b Linfen People's Hospital; c Guangzhou University of Chinese Medicine, Guangzhou, China.

**Keywords:** Linggui Zhugan Decoction, peripheral vertigo, protocol, systematic review

## Abstract

**Background:**

Vertigo is a sense of movement or rotation of the patient's own or an external object. At present, western medicine treatment such as vestibular suppressant medications commonly used in clinical practice are ineffective and have adverse reactions. In traditional Chinese medicine, Linggui Zhugan Decoction (LZD) was used by doctors to warm yang for resolving fluid retention, strengthen the spleen and clear away dampness, with significant effect. Recently, some clinical studies have also shown that LZD has reliable effect in treating peripheral vertigo, but there is no systematic evidence. Therefore, this study aims to objectively evaluate the efficacy and safety of LZD in the treatment of peripheral vertigo.

**Methods:**

Eight electronic databases will be searched from inception to August 2020 by 2 independent researchers, in order to collect qualified randomized controlled trials (RCTs) on the LZD treatment for peripheral vertigo. The therapeutic effects according to Clinical efficacy will be adopted as the primary outcomes. RevMan V.5.3 software will be used for the data synthesis and the Cochrane's risk of bias assessment tool will be used to assess the risk of bias.

**Results:**

This review will conduct a high-quality synthesis on present evidence of LZD for peripheral vertigo.

**Conclusion:**

The conclusion of the study will indicate whether LZD is an effective treatment for peripheral vertigo by providing updated evidence.

**PROSPERO registration number:**

PROSPERO CRD 42021238817.

## 1 Introduction

Vertigo is one of the most familiar chief complaints in the department of neurology, presenting as a sense of movement or rotation of the patient's own or an external object,^[1]^ it can be divided into peripheral vertigo and central vertigo.^[2]^ Peripheral vertigo refers to vertigo caused by the vestibular receptors lesions in the inner ear and the extracranial vestibular lesions in the inner auditory canal,^[3]^ usually including Meniere's disease, benign paroxysmal positional vertigo (BPPV), drug-induced vertigo, vestibular neuronitis and labyrinthitis.^[4–7]^ According to the data from large population-based studies, from about 15% to over 20% of adults suffer from vertigo yearly, among which peripheral vertigo is accounted for a significant part.^[8]^ As a result of vascular lesions, local inflammation, poisoning, trauma and other factors damaging vestibular function, peripheral vertigo patients are often accompanied by severe vertigo, hearing changes, and autonomic nervous symptoms and other unbearable pain.^[9]^ Therefore, it is necessary to seek for an effective treatment.

Vestibular suppressant, antihistamines, minimal invasive interventions, plugging of the semicircular canal, labyrinthectomy, and neurectomy are routinely used in clinical practice to treat peripheral vertigo. However, these interventions are more or less ineffective or have other adverse effects such as damage to the cochlea and the vestibular organs.^[10–12]^

There are a number of studies show that traditional Chinese medicine has unique advantages in reducing the symptoms and improving prognosis of the patients with peripheral vertigo.^[13–15]^ Linggui Zhugan Decoction (LZD) is a classic formula in Synopsis of Golden Chamber, containing Fuling (Poria), Guizhi (Ramulus Cinnamomi), Baizhu (Atractylodes macrocephala), and Gancao (Glycyrrhiza uralensis), which is traditionally applied to warm yang for resolving fluid retention, strengthen the spleen and clear away dampness. A relevant clinical observation has proved that LZD combined with other drugs is effective in treating Meniere's disease and can efficaciously prevent vertigo attacks.^[16,17]^

What is more, LZD has shown significant efficacy in many physicians’ clinical practice experience in the treatment of peripheral vertigo, and also there are certain means of western medicine combined treatment. However, due to the complexity of the peripheral vertigo itself and the scattered clinical experience, there is a lack of systematic evidence which is strong enough to support, influencing the popularization and application of this therapy to a certain extent. Therefore, aiming to objectively evaluate the efficacy and safety of LZD in the treatment of peripheral vertigo, this study intends to collect randomized controlled trial of LZD in the treatment of peripheral vertigo, performing a systematic review and meta-analysis, and provide a reliable evidence-based basis for clinical application.

## 2 Methods

### 2.1 Protocol design and registration

This protocol sternly complies with the the Preferred Reporting Items for Systematic Reviews and Meta-Analyses Protocols (PRISMA-P) principles. And it has been registered on PROSPERO (CRD 42021238817). We will wield Cochrane Handbook about Systematic Reviews of Interventions to implement the study.^[18]^

### 2.2 Inclusion criteria

#### 2.2.1 Study categories

All the appropriate randomized controlled trials (RCTs) that apply LZD to treat peripheral vertigo will be included, whether blind method or allocation concealment is used or not, while the language will be confined in English and Chinese.

#### 2.2.2 Study participants

Those patients who are definitely diagnosed with peripheral vertigo will be involved, irrespective of age, gender, race, region or education.

#### 2.2.3 Study interventions

The matched group was under the pure western medicine treatment without the limitation on the types, dose or course of treatment. The control group was treated with LINGGUI ZHUGAN DECOCTION, including LINGGUI ZHUGAN DECOCTION, LINGGUI ZHUGAN DECOCTION boned with western medical treatment and LINGGUI ZHUGAN DECOCTION plus or minus, which also has no restriction on formulation, dose or course of treatment.

#### 2.2.4 Outcome indicators

##### 2.2.4.1 Clinical efficacy

That vertigo and other symptoms basically disappeared after treatment, returning to normal study and work life can be regarded as recovery; vertigo was significantly reduced, the number of attacks decreased >60%, dizziness or dizziness and other clinical symptoms were significantly reduced, no self or scenery rotation, shaking, normal life and work for obvious effect; vertigo was significantly reduced, the attack frequency was reduced by >60%, there was a slight sense of rotation and shaking of the self or scenery, other clinical symptoms were slightly improved, work and life were affected to some extent to be effective; vertigo and other clinical symptoms do not improve or worsen indicating that it is invalid.^[19]^

##### 2.2.4.2 Vertigo disorder

Employ dizziness handicap inventory (DHI) to rate vertigo disorders before and after treatment in both groups, including the comprehensive state of the patient's body, function and mood, the total score is 100 points, the higher the score indicates the vertigo disorder is more obvious.^[20]^

##### 2.2.4.3 Adverse reactions

Adverse reactions occurred during treatment, such as nausea, vomiting and rash, were recorded in both groups. Adverse reaction incidence = number of adverse reactions/total number of cases × 100%.^[20]^

### 2.3 Exclusion criteria

1. Non-RCT study;
2. The intervention measures did not include the intervention measures mainly for LZD;
3. Literature with incomplete data;
4. No evaluation index;
5. Animal and cell basic research, etc.

### 2.4 Search strategy

Computer retrieve the literature, whose publication ended September 24,2020, from Pubmed, Cochrane, Embase, Web of Science, China National Knowledge Infrastructure, China Biological Medicine, Chongqing VIP, and Wan-fang databases, with words “LINGGUI ZHUGAN decoction” “FULING” “peripheral vertigo” “GUIZHI” “GANCAO” “BAIZHU” as retrieval. Take Pubmed database as an example, the retrieval strategy of literature will be shown in Supplement 1.

### 2.5 Data collection and analysis

Data collection and analysis will be carried out independently by 2 professionally trained researchers. In case of disagreement, the opinions of a third researcher are asked and a common understanding is reached.

#### 2.5.1 Literature screening

**Figure 1:**
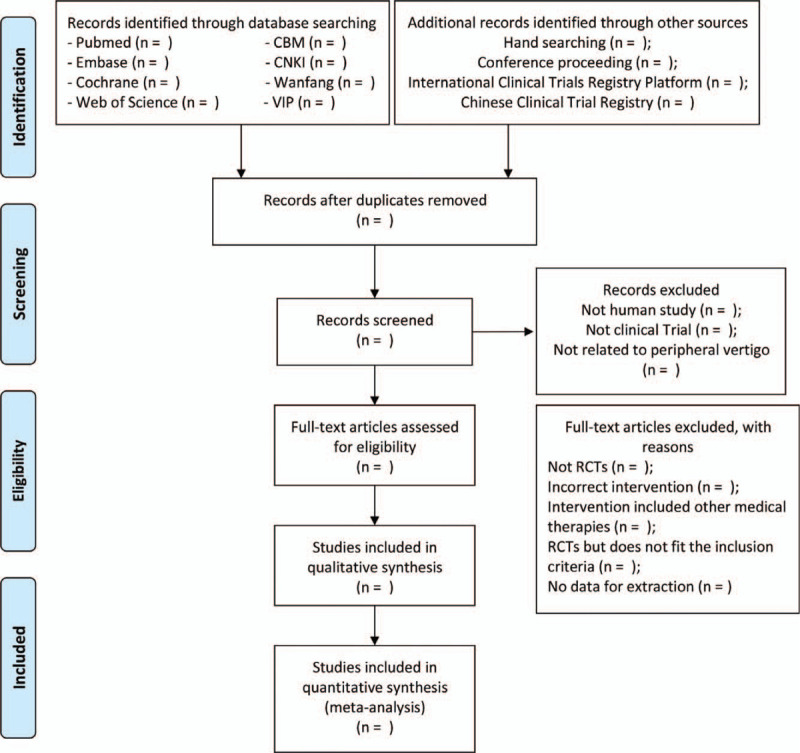
Preferred Reporting Items for Systematic Reviews and Meta-Analyses (PRISMA) flow chart of study selection process.

We will utilize NoteExpress and Endnote to manage the literature, which will be used to eliminate the duplicate literature at first. Then the literature was initially screened by reviewing the titles and abstracts of the literature, to judge whether the literature types, participants and intervention measures meet the criteria for inclusion in this meta-analysis, excluding the unqualified literature. Then, read and evaluate the selected literature that may meet the requirements, so as to determine whether the literature meets the inclusion criteria of meta-analysis. The literature screening flow chart is shown in Fig. 1.

#### 2.5.2 Data extraction and management

We will use the predesigned data extraction table to comprehensively and systematically collect the following information:

1. General information (the number, title, corresponding author and contact information of the included study);
2. Participants’ characteristics (age, sex and course of disease);
3. Intervention measures (drug names, drug delivery route, dose and course of treatment);
4. Outcome indicators (clinical efficacy, vertigo symptoms and adverse reactions).

#### 2.5.3 Dealing with missing data

For studies with incomplete data, we will contact the original author via email to obtain complete research data. If complete data is not available, the study data is excluded.

### 2.6 Literature quality evaluation

According to the “Risk of bias Assessment” tool recommended by the Cochrane Collaboration, the assessment includes the following 6 aspects:

1. Random allocation;
2. Allocation scheme hiding;
3. Whether blind method is adopted;
4. Integrity of the resulting data;
5. Selective reporting of research findings;
6. Bias from other sources.

Two researchers independently made the judgment of “high risk,” “low risk” and “unclear” on the above 6 items with all the included study data, and then cross-checked after completion. In case of differences, we will discuss and decide together with the third researcher. The risk assessment bias graph for this study will be generated using Review Manager 5.3.

### 2.7 Statistical analysis

#### 2.7.1 Selection of effect index

Apply Review Manager 5.3 software to statistics and analysis the data. Different data types correspond to different effect indexes. In this study, relative risk (RR) will be the effect index of dichotomous data, while weighted mean difference (WMD) for continuous variables if the measuring units are the same, or standard mean difference (SMD) if the units of measurement are different. We will use the effect value and 95% confidence interval as comparison. The heterogeneity among the included studies should be tested, which is depending on *X*^2^ and *I*^2^. If *P* > .1, *I*^2^ ≤ 50%, the studies will be considered to be homogeneous, so the Fixed model will be used for meta analysis; if *P* ≤ .1, (3tI2 >50%, it refers that studies involved have heterogeneity, and we need to do subgroup analysis, sensitivity analysis and choose Random model to solve the problem.

#### 2.7.2 Subgroup analysis

When there exists heterogeneity, do the subgroup analysis of factors which may affect the end index. Meta analysis will be carried out respectively in the group treated with LZD alone and the group treated with LZD combined with western medicine; subgroup analysis will be conducted according to LZD with different dosage forms, different courses of treatment and different western medicine.

#### 2.7.3 Sensitivity analysis

To test the stability and reliability of meta-analysis results, we will do the sensitivity analysis. Once different conclusion appear in the process, it indicates that we must be more careful in the interpretation and conclusion.

#### 2.7.4 Publication bias

If the number of documents included reach 10 or more, we will use Review Manager 5.3 to generate “inverted funnel” graph, which is use for evaluating publication bias.

#### 2.7.5 Evidence quality evaluation

The recommended Assessment, Development and Evaluation (GRADE) criteria developed by the World Health Organization and international organizations will be applied to assess the quality of evidence in 5 areas (risk of bias, consistency, directness, accuracy and publication bias), with the quality of the evidence divided into high, medium, low and very low.

### 2.8 Ethics

As the data source of this study is from the literature of major databases, it does not involve the recruitment of patients or the collection of personal information, so the sanction of the ethics committee is unnecessary.

## 3 Discussion

In recent years, people pay more and more attention to peripheral vertigo. Due to vascular lesions, local inflammation, poisoning, trauma and other factors lead to vestibular sensory organs and vestibular nucleus after the vestibular nerve conduction fiber lesions, often make patients need to endure unbearable pain, greatly affecting their lives. At present, there are many methods to treat peripheral vertigo, but most of them need surgery, which is easy to cause secondary injury to patients.

The traditional Chinese medicine pathological mechanism of peripheral vertigo is nothing more than wind, phlegm and deficiency. It is often treated with hidden yang and resolving phlegm, tonifying the kidney and invigorating the spleen in order to nourish it.^[21]^

LZD is a classic prescription in the outline of Synopsis of Golden Chamber, containing Fuling (Poria), Guizhi (Ramulus Cinnamomi), Baizhu (Atractylodes macrocephala), and Gancao (Glycyrrhiza uralensis). It is traditionally used to warm yang and hydrophobic, invigorate the spleen and remove dampness. It is an effective prescription for the treatment of peripheral vertigo. In the prescription, Poria cocos is reused as the king medicine, invigorating the spleen and promoting diuresis, permeating and removing water, which can not only resolve phlegm, but also reduce the adverse effect of moisture and qi. Compatible with spicy and warm cassia twig, the function of warming yang and transforming qi, Pingchong down adverse. Atractylodes macrocephala can invigorate spleen dryness and dampness. Licorice in this prescription, first, together with Cinnamomum cassia twig, can warm and tonify Zhongyang; second, it can replenish qi and invigorate the spleen together with Atractylodes macrocephala Koidz to make water; third, it can reconcile various medicines. All convenience plays the role of warming yang and invigorating the spleen, light osmosis and dampness, which is suitable for the basic pathogenesis of phlegm-drinking peripheral vertigo with deficiency of middle yang.

Modern pharmacological studies have found that LZD can effectively relieve the symptoms of vertigo, reduce the level of serum hypersensitive C-reactive protein, effectively accelerate the blood flow velocity of vertebral artery and basilar artery, and the curative effect is better than western medicine.^[22]^ The effective components of cassia twig can promote blood circulation, relieve limb spasm, dilate blood vessels, improve blood circulation, and increase blood flow in the brain.^[23]^ Atractylodes macrocephala Koidz can dilate blood vessels, increase cerebral blood flow, regulate craniocerebral blood circulation, and diuresis, anticoagulation, inhibit thrombosis and so on.^[24]^

Through this study, we can systematically evaluate the efficacy and safety of LZD in the treatment of peripheral vertigo, and understand the difference between LZD and western medicine in the treatment of peripheral vertigo. And the efficacy of LZD combined with western medicine in the treatment of peripheral vertigo is better than that of western medicine alone. In addition, we can understand the adverse reactions of LZD, which is beneficial to clinical application. However, this study also has some limitations, such as less literature, small sample size and so on. In addition, due to the use of LZD in the treatment group, rather than the original prescription of LZD, the dosage is different, there is a certain heterogeneity. In addition, this study only searches English and Chinese literature, and may ignore the research or reports in other languages, which has a certain publication bias. Therefore, high-quality, large-sample literature support is still needed to improve the reliable basis for clinical and patients to use LZD in the treatment of peripheral vertigo.

## Author contributions

**Conceptualization:** Wanning Lan, Jiyuan Zheng, Danyun Li, Xinju Hou.

**Data curation:** Wanning Lan, Jiyuan Zheng, Danyun Li, Ziyin Chen.

**Formal analysis:** Jiyuan Zheng, Danyun Li.

**Investigation:** Hongmei Ma.

**Methodology:** Hongmei Ma.

**Software:** Liang Guo.

**Supervision:** Hongmei Ma, Xinju Hou.

**Validation:** Yong Chen.

**Writing – original draft:** Hongmei Ma, Liang Guo, Yong Chen.

**Writing – review & editing:** Hongmei Ma, Liang Guo, Yong Chen.

## Supplementary Material

Supplemental Digital Content
