# Supplementary material for: Linggui Zhugan Decoction for peripheral vertigo: A protocol for systematic review and meta-analysis
Source: Medicine (Baltimore). 2021 Apr 23;100(16):e25563. doi: 10.1097/MD.0000000000025563 (PMC8078309; doi:10.1097/MD.0000000000025563)
Supplement: Supplemental Digital Content [file medi-100-e25563-s001.doc]

**Supplement 1. Search strategy used in PubMed database**

| Number | Search Terms |
| --- | --- |
| #1  #2  #3  #4  **#5**  #6  #7  #8  #9  #10 | peripheral vertigo[MeSH Terms]  peripheral vertigo[Title/Abstract]  peripheral[Title/Abstract])AND(vertigo[Title/Abstract])  #1 OR #2 OR #3  LINGGUI ZHUGAN DECOCTION[MeSH Terms]  (((FULING[MeSH Terms]) AND (GUIZHI[MeSH Terms])) AND (BAIZHU[MeSH Terms])) AND (GANCAO[MeSH Terms])  LINGGUI ZHUGAN DECOCTION[Title/Abstract]  (((FULING[Title/Abstract])AND(GUIZHI[Title/Abstract])) AND(BAIZHU[Title/Abstract]))AND(GANCAO[Title/Abstract])  #5 OR #6 OR #7 OR #8  #4 AND #9 |
